# How Parenting Styles Link Career Decision-Making Difficulties in Chinese College Students? The Mediating Effects of Core Self-Evaluation and Career Calling

**DOI:** 10.3389/fpsyg.2021.661600

**Published:** 2021-05-19

**Authors:** Xiaoyan Tian, Bijuan Huang, Hongxia Li, Shaowen Xie, Komal Afzal, Jiwei Si, Dongmei Hu

**Affiliations:** School of Psychology, Shandong Normal University, Jinan City, China

**Keywords:** parenting styles, core self-evaluation, career calling, career decision-making difficulties, gender difference

## Abstract

The purpose of this study was to explore the relationship between parenting styles and career decision-making difficulties in college students, and uncovered the mediating roles of core self-evaluation and career calling. A total of 1,127 undergraduates were recruited to complete the questionnaires about parenting styles, core self-evaluation, career calling, and career decision-making difficulties. The results showed that: (1) Positive and negative parenting styles could positively predict career decision-making difficulties in college students. (2) Core self-evaluation and career calling mediated the relationship between parenting styles and career decision-making difficulties. Sequential dual mediators only found in which positive paternal and maternal parenting styles predict career decision-making difficulties through core self-evaluation and career calling. (3) Further analysis revealed gender difference in the relationship between parenting styles and career decision-making difficulties. The relation between paternal positive parenting style and career decision-making difficulties was significant in male students, but absent in female students; the relation between maternal positive parenting and career decision-making difficulties and the relation between paternal negative parenting and career calling were significant in female students, but absent in male students; and the relation between career calling and career decision-making difficulties was greater in male than in female. The current study expanded and deepened those existing understandings about the relationship between parenting styles and adolescents’ career decisions, so as to further reveal its internal mechanism and provide more reasonable suggestions and targeted guidance for career counseling.

## Introduction

Recently, the employment pressure is increasingly severe under the situation of saturated job market (Wang, 2013). Until now, the number of new university fresh graduates has been climbing, and the number of graduates is constantly at a record high every year. In 2020, the number of university fresh graduates is close to 9 million, and a large number of graduates flow into the market and compete together for the remaining jobs, which is naturally very fierce competition. As a result, the employment pressure on graduates is becoming increasingly severe. Choosing a suitable career and successfully entering the workplace is an important life issue for university students. College students are at the stage of transition from “student” to “professional.” When faced with the first career decision in their lives, due to the influence of subjective and objective factors, such as their own lack of preparation, lack of information about the external environment, college students are unable to make a wise career decision, that is, they will face difficulties in career decision-making, which leads to the social phenomenon of college students’ difficulties in employment (Yang et al., 2020). The university stage is the fundamental period of career preparation. As an essential turning point in the process of individual socialization, successful career decisions are an important way to integrate into society. Career decision-making difficulties which become one of prominent issues in the growth of college students have attracted scholarly attention (Fabio et al., 2012; Shen and Gu, 2018). Career decision-making difficulties refer to the difficulty an individual faces in making a final decision during the process of career choice (entry stage or career change). It denotes individuals’ difficulty of choosing one of several careers or knowing what occupation to pursue (Long and Peng, 2000). If they can solve many difficulties of career decision making during university stage successfully, they will choose a suitable career for themselves and enter job market successfully with individuals’ rational career decisions. Consequently, it has great practical significance to study the career decision-making difficulties of college students.

### The Relations Between Parenting Styles and Career Decision-Making Difficulties

Researches showed that individuals’ career decision-making was closely related to environmental factors, such as family (e.g., Fulya et al., 2010; Bratcher, 2011; Liu, 2020) and social context (e.g., Bright et al., 2005). As the first place of individual socialization, it is crucial for researcher to explore the relationship between family factors and career decision-making. The results of studies on the career development process of university students from the perspective of family systems theory confirm that good interaction between family members influences the career development of university students to a certain extent. Among many family factors, parenting styles have been widely concerned by many scholars. Family parenting style refers to the way parents treat their children in the process of raising them and the way they educate them, which plays a pivotal role in the growth and development of individuals (Wu et al., 2020). Darling and Steinberg (1993) defined parenting styles as corresponding to the emotional atmosphere in which parents raise and educate children, and it is also characterized by the dimensions of responsiveness and demands, and shows the nature of parent-child interaction. As Roe (1956) pointed out that different parenting styles could influence individual career decision-making process in his parental influence theory of career decision-making. He emphasized that overprotection and refusal were more likely to lead to career decision-making difficulties, and the acceptance and warmth of parenting styles could improve self-confidence, so that individuals choose career path more smoothly. Besides, recent researches supported the significance of parenting styles on career decision-making (Blustein, 2011; Luo, 2020). According to Liu (2020), parenting styles are closely related to children’s career decisions, and parents who want to improve their children’s self-efficacy in career decisions can start with positive parenting styles. Further research found that, a positive relationship existed between the parental authoritarian style and career decision-making difficulties (Koumoundourou et al., 2011). Liu (2008) found that the warmth of parents gave college students a feel of high level career decision-making efficiency, which helped individuals to ease career decision-making difficulties, improve and enhance their career decision-making level. Parental warmth had a stable and lasting indirect effect on career decision-making self-efficacy through the sense of responsibility in college students, and then promoted college students’ career decision-making efficacy (Hou et al., 2013). Furthermore, neglectful and indulgent parenting styles were associated with higher scores on a measure of career decision-making difficulties (e.g., Sovet and Metz, 2014). That means negative parenting styles do not support children in their career decisions (Fulya et al., 2010).

Although these findings consistently showed that parenting styles are an effective predictor of career decision-making difficulties, there is still a lack of research on the mechanism underlying this relationship. Thus, it is imperative to expand the previous research and examine the intermediate variables (core self-evaluation and career calling) between parenting styles and career decision-making difficulties among college students. Arindell et al. (1999) classify parenting styles into three categories: rejection, emotional warmth and overprotection. Rejection and overprotection are negative parenting styles and emotional warmth is a positive parenting style. Therefore, this study will look at two aspects of positive parenting styles and negative parenting styles.

### The Potential Factors Mediated the Link Between Parenting Styles and Career Decision-Making Difficulties

Previous studies showed that the predictive effect of parenting styles on career decision-making was influenced by some internal factors, such as core self-evaluation (e.g., Koumoundourou et al., 2011), self-efficacy (e.g., Lease and Dahlbeck, 2009), and sense of responsibility (e.g., Hou et al., 2013). In the family, parents’ behavior, temper, and parenting styles will unconsciously affect children’s behavior standards (e.g., Xie, 2012; Yang et al., 2017). At the same time, parenting styles will also affect the children’s behavior through personal internal factors, such as personality, emotion and so on (Hou et al., 2013). Bandura (1986) proposed social cognitive theory, prescribing that environmental factors could act on individual behavior through human internal factors. Thus, we can predict that parenting styles further influences career decision-making difficulties by affecting individuals’ personalities and emotions. It is of theoretical and practical significance to probe into the internal mechanism of influencing college students’ career decision making from the perspective of internal factors.

Core self-evaluation is one of the most important personality factors. Core self-evaluation, as a cognitive factor, refers to an individual’s most basic evaluation of his or her own abilities and values (Chen et al., 2020). As a typical individual factor, core self-evaluation is closely linked to various functions of human beings, such as cognition (e.g., Peng and Cao, 2020) and action (e.g., Lv and Zhou, 2019), and the relationship with parenting styles has been confirmed by various researches. First, Yang et al. (2017) proposed that core self-evaluation in high school students played an important mediating role between parenting styles and self-regulated learning; Through a study of college students’ family parenting styles and core self-evaluation, Zhang (2016) found that college students’ family parenting styles and core self-evaluation were significantly related. Second, previous research also supported the role of core self-evaluation in relation to career decision-making, suggesting that enhancing core self-evaluation likely helps individuals make a career exploration and decision (Koumoundourou et al., 2011; Fabio et al., 2012; Hou, 2017). Xie (2012) proved that warmth of parents predict college students’ core self-evaluation positively. Parental support and understanding could promote good self-awareness, and then will be more spontaneous and active in career exploration and thinking about suitable career choices, so that they can have a clear understanding of their career planning (Liu and Zhang, 2017). This is in line with Super’s (1968) career development theory to a certain extent, that emphasized personality and initiative. Thus the current study posits that core self-evaluation likely acts as the first mediator following parenting style.

Following core self-evaluation, career calling could act as the second mediator proximal to career decision-making difficulties. Career calling is a dynamic process that takes on different states (searching a calling, perceiving a calling, living a calling) as life experiences change (Duffy and Dik, 2013). Searching a calling is the process by which individuals intentionally explore, experience and even develop a sense of call for themselves. Perceiving a calling refers to the individual’s perception that he or she is called or summoned to a specific occupation or activity and involves the existence of a calling, i.e., the individual finally knows where his or her inner calling is directed. Searching a calling and perceiving a calling are not the end-state of calling, but the real commitment to the realm where directed to achieve the calling is the goal (Tian and Zuo, 2014). This study use searching a calling as the research object. Career calling, which denotes a strong and meaningful passion experienced by individuals working in a particular field, has included a positive and passionate experience of work (Dobrow and Tosti-Kharas, 2011; Zhang, 2015). Previous studies have shown that individuals with high core self-evaluations put their careers in a positive framework and have a positive perception of job characteristics and values (Judge et al., 1997; Judge and Larsen, 2001). Further research also found that core self-evaluation has a positive effect on career calling, while core self-evaluation negatively moderates the positive relationship between career calling and its positive outcomes (e.g., life satisfaction) (Duffy et al., 2012). This also verifies the existentialist career theory to a certain extent, the individual’s self-concept helps to improve the understanding of the meaning and goal of life, so as to stimulate the individual’s career calling (Liu, 2018). Therefore, high core self-evaluation may be associated with high sense of career calling.

Individuals with a high sense of career calling hope that their work can make a valuable contribution to society, and experience inner pleasure and self-realization in their work (Hall and Chandler, 2005; Neubert and Halbesleben, 2015). Career calling has been found to be related to various aspects of future career. Dik et al. (2009) pointed out that career calling can promote the clarity of job-hunting, and the students with high sense of career calling have a clearer understanding of career goals and future career planning. Meanwhile, career calling also has a significant impact on employability and career exploration (Cheng et al., 2017). More importantly, previous study has also showed that college students’ career calling can predict the degree of career decision-making positively, and negatively predict career decision-making difficulties (Duffy and Sedlacek, 2007). Thus, students with low levels of core self-evaluation are prone to negative academic emotions, which in turn cause difficulties in career decision-making (Zhang, 2020). Combined with previous studies and social cognitive theory, core self-evaluation and career calling may play a chain-mediated role in the process of parenting styles predict career decision-making difficulties.

### Gender Differences

There was obvious gender difference, however, in career decision-making difficulties. Specifically, Researches have showed that gender differences emerged in emotional and personality-related factors in career decisions, not in cognitive factors (e.g., Gati et al., 2012). Male participants generally experienced fewer difficulties than female participants in career decision-making (Zhou and Santos, 2007; Gadassi et al., 2015). That maybe because female had fewer opportunities to choose a career and generally perceived more difficulties due to social factors (Lv, 2010). At the same time, studies have concluded that there were significant gender differences in core self-evaluation (Xie, 2012). Decision-making difficulties in male were not affected by core self-evaluation. In contrast to male, core self-evaluation completely mediated the relationship between autocratic style and decision-making difficulties in female (Koumoundourou et al., 2011). In addition, the degree of perception of difficulty in making career decisions was higher for female than for male, and higher in liberal arts than in science (Lv, 2010). Self-construction and self-improvement have important connections with significant others, especially parents. Women are more inclined to learn information from significant others to construct themselves, while men are more inclined to construct themselves independently (Gardner and Gabriel, 1999). Thus, we can predict that compared with male, female are more deeply affected by their parents in terms of self-evaluation, and parenting styles can have a more profound impact on the mediating effect of core self-evaluation and career calling on career decision-making difficulties, which has not been explored by previous studies. This study further examined the moderation of gender on the link between parenting styles and career decision-making difficulties.

### The Present Study

The current study examined the important prediction of parenting styles for distal career decision-making difficulties in college students, and further revealed the internal mechanism. The study hypothesized a sequential dual mediator model in which core self-evaluation and career calling sequentially function as mediators in the prediction of parenting styles for career decision-making difficulties. Specifically, this study hypothesized that: (1) positive/negative parenting styles could negatively/positively predict career decision-making difficulties; (2) parenting styles could also influence career decision-making difficulties through core self-evaluation and career calling; (3) gender differences existed in the process of parenting styles to predict career decision-making difficulties through core self-evaluation and career calling. It will help researchers to understand the internal and external causes of career decision-making difficulties for college students.

## Materials and Methods

### Participants

A total of 1,240 juniors and seniors were recruited from five public universities in Jinan, China. The questionnaires were released on the Internet, which was freely filled in by qualified subjects with the consent of the teacher and the students. 113 participants were excluded due to responding sloppily or in sequence. The effective rate of date analyzed was 90.89%. Of the sample, this study included 47.2% male, and 59.6% were junior. All of the students received a pen as compensation for their participation.

### Instruments

#### Parenting Styles

Short-Egna Minnenav Barndoms Uppfostran for Chinese (s-EMBU-C) (Jiang et al., 2010), was adapted to measure parenting styles. This questionnaire consisted of 21 items from father and same items from mother. In the present study, rejection and overprotection were defined as a negative parenting style, while emotional warmth was a positive parenting style. Thus, there were four facets in this study to exam parenting styles: paternal positive parenting style, paternal negative parenting style, maternal positive parenting style, and maternal negative parenting style. Participants rated each item on a 4-point Likert scale ranging from 1 (never) to 4 (always). In the present study, Cronbach’s α coefficient of four facets of the scale ranged from 0.87 to 0.91.

#### Core Self-Evaluation

The Core Self-Evaluation Scale (Judge et al., 1997; Ren and Ye, 2009) was used to evaluate the level of core self-evaluation level. This scale is primarily related to self-esteem, general self-efficacy, psychological control source, and emotional stability. Participants were invited to rate items on a 5-point Likert scale ranging from 1 (strongly opposed) to 5 (strongly agreed). The scale consists of 8 items, Lower scores indicated a higher level of core self-evaluation. In the present study, the Cronbach’s α coefficient of this scale was 0.82.

#### Career Calling

The Calling Scale (Zhang, 2015) was used to measure the level of career calling. This scale includes three dimensions of altruistic contribution, guiding force and meaning, and value. The scale consists of 8 items participants rated each item on a 5-point Likert scale ranging from 1 (totally inconsistent) to 5 (totally consistent). Higher scores indicated a stronger endorsement. The Cronbach’s α coefficient of the scale in the present study was 0.87.

#### Career Decision-Making Difficulty

Career decision-making difficulties were measured by the Career Decision-Making Difficulties Questionnaire (Du and Long, 2006). There are 22 items in this scale, belonging to the four dimensions, that is information exploration, self-exploration, planning exploration, and goal determination. Participants rated each item on a 5-point Likert scale ranging from 1 (very inconsistent) to 5 (very consistent). Higher scores indicated a lower level of career decision-making difficulties. In the present study, the Cronbach’s α coefficient of the scale was 0.91.

#### Data Analysis

SPSS 22.0 and AMOS 24.0 were used to analyze the data, including descriptive statistical analysis and correlation analysis. *T*-test was performed to explore gender differences. In order to further determine gender differences in the mediating effect of career calling and core self-evaluation on parenting styles and career decision-making difficulties, multi-group comparison analysis was performed.

Structural equation modeling analysis was conducted separately for male and female using AMOS 24.0. In the unconstrained model, all parameters were allowed to vary across groups, whereas in the constrained model, all path coefficients were fixed as equal across groups. A better model fit in the unconstrained model indicates that the relations between parenting styles and career decision-making difficulties differ across the two groups.

Model fit of all the structural equation models was assessed by the comparative fit index (CFI), the Tucker-Lewis Index (TLI), the root-mean-square error of approximation (RMSEA), and the chi-square statistic. Because the chi-square statistic is sensitive to large samples, model fit indices were used as the primary criteria to evaluate model fit. The TLI and the CFI range between 0 and 1, with values above 0.90 indicating adequate model fit (Hu and Bentler, 1999). A rule of thumb for the RMSEA is that values ≤ 0.05 indicate close approximation, values between 0.05 and 0.08 indicate reasonable error of approximation, and values ≥ 0.10 indicate poor fit (Browne and Cudeck, 1992). The chi-square test of difference was used to compare the fit of the nested models. A significant chi-square test of difference suggests that the less constrained model should be retained, whereas a non-significant test indicates that the two models provide equal fit to the data.

## Results

### Preliminary Analyses

Table 1 summarizes the means, standard deviations, and correlations of the variables. It shows a general pattern of relatedness among the variables except that the career decision-making difficulties are uncorrelated with paternal negative parenting style and maternal negative parenting style (*p*s > 0.05). In order to explore whether gender moderated the links between parenting styles and career decision-making difficulties, independent sample *T*-test and Pearson correlation analysis was conducted on the variables in male and female samples, respectively (see Table 2). The result showed that there were significant gender differences in those variables except paternal positive parenting style and career decision-making difficulties. Meanwhile, Pearson correlation analysis showed a general pattern of relatedness among the variables (*p*s < 0.05) except that the paternal negative parenting style was uncorrelated with career decision-making difficulties (*r* = –0.05, *p* > 0.05) in male sample, the paternal and maternal parenting style were uncorrelated with career decision-making difficulties (*p*s > 0.05) in female sample.

**TABLE 1:** Descriptive statistics, correlation analysis, and gender difference of variables.

Variable	1	2	3	4	5	6	7	8
1. Gender	–							
2. FPS-N	−0.25**	–						
3. FPS-P	0.04	−0.13**	–					
4. MPS-N	−0.21**	0.73**	−0.13**	–				
5. MPS-P	0.11**	−0.24**	0.64**	−0.23**	–			
6. CSE	0.06*	0.12**	−0.27**	0.20**	−0.24**	–		
7. CC	0.09**	−0.13**	0.25**	−0.12**	0.31**	−0.19**	– –	
8. CDD	0.01	–0.02	0.31**	–0.05	0.28**	−0.32**	0.47**	–
M	–	24.47	19.29	25.66	21.22	21.45	40.45	55.42
SD	–	6.70	4.64	6.87	4.69	5.17	6.56	9.56

FPS-N, negative paternal parenting style; FPS-P, positive paternal parenting style; MPS-N, negative maternal parenting style; MPS-P, positive maternal parenting style; CSE, core self-evaluation; CC, career calling; CDD, career decision-making difficulties; N = 1127. *p < 0.05; **p < 0.01 (same below).

**TABLE 2:** Fitting indexes of model in boys and girls.

	χ^2^	RMSEA	NFI	GFI	Δχ2	Δdf	ΔNFI	ΔIFI
M_*male*_	223.33	0.073	0.95	0.94	–	–	–	–
M_*Female*_	229.48	0.071	0.93	0.95	–	–	–	–
M_1_	452.81	0.051	0.94	0.95	–	–	–	–
M_2_	479.42	0.050	0.94	0.94	26.61	9	0.004	0.004
M_3_	509.52	0.049	0.93	0.94	30.10	13	0.004	0.004

**M*_*male*_, *Male Model; M_*Female*_, Female Model; M1, Unconstraint model; M2, Model with equal measurement weight; M3, Model with equal structural weight.**

### Structural Equation Modeling

Based on previous studies and the analysis of the relationship among variables in this study, the mediating role of core self-evaluation and career calling in the relationship between parenting styles and career decision-making difficulties was investigated. A multi-mediation model was performed by taking the four dimensions of parenting styles as predictive variables, core self-evaluation and career calling as mediating variables, and career decision-making difficulties as outcome variables. The results showed that the model fitted well (χ^2^/*df* = 7.04, RMSEA = 0.07, CFI = 0.95, TLI = 0.93).

**FIGURE 1:**
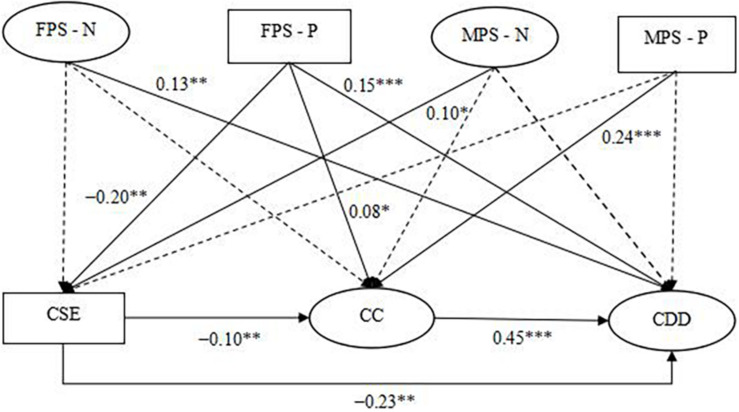
The mediating path of parenting styles and career decision-making difficulties. The solid line means the path coefficient is significant, and the dotted line means the path coefficient is not significant, same below. FPS-N, negative paternal parenting style; FPS-P, positive paternal parenting style; MPS-N, negative maternal parenting style; MPS-P, positive maternal parenting style; CSE, core self-evaluation; CC, career calling; CDD, career decision-making difficulties.

The results showed that core self-evaluation substantially predicted career calling (β = – 0.10, *p* < 0.01)and career decision-making difficulties (β = –0.23, *p* < 0.001), career calling positively predicted career decision-making difficulties (β = 0.45, *p* < 0.001) (see Figure 1). Positive paternal parenting style had significant predictive effects on core self-evaluation (β = –0.20, *p* < 0.001), career calling (β = 0.08, *p* < 0.05) and career decision-making difficulties (β = 0.15, *p* < 0.001), indicating that core self-evaluation and career calling played a mediating role between positive paternal parenting style and career decision-making difficulties. The indirect effect was 0.044, accounting for 38.94% of the total effect (see Table 3 and Figure 1); Although the direct effect of negative paternal parenting style on career decision-making difficulties was significant (β = 0.13, *p* < 0.01), it had no significant predictive effect with core self-evaluation and career calling (*p*s > 0.05); Positive maternal parenting style was not found to predict career decision-making difficulties (*p* > 0.05), but it was found to (positively) predict career calling (β = 0.24, *p* < 0.001), indicating that positive maternal parenting style indirectly affected career decision-making difficulties by college students’ career calling. The indirect effect was 0.051, accounting for 75% of the total effect (see Table 3 and Figure 1). Negative maternal parenting style was not found to predict career calling and career decision-making difficulties (*p*s > 0.05), but it was found to (positively) predict core self-evaluation (β = 0.10, *p* < 0.05), indicating that negative maternal parenting style indirectly affected career decision-making difficulties by college students’ core self-evaluation and career calling. The indirect effect was –0.017, accounting for 41.47% of the total effect (see Table 3 and Figure 1). To sum up, there were multiple mediators between parenting styles and career decision-making difficulties.

**TABLE 3:** Indirect effect.

Path	Indirect effect	Percentage of total effect
FPS-P→CSE→CC→CDD	0.004	3.54%
FPS-P→CSE→CDD	0.022	19.47%
FPS-P→CC→CDD	0.017	15.04%
MPS-N→CSE→CC→CDD	–0.003	7.32%
MPS-N→CSE→CDD	–0.014	34.15%
MPS-P→CC→CDD	0.051	75.00%

**FPS-P, positive paternal parenting style; MPS-N, negative maternal parenting style; MPS-P, positive maternal parenting style; CSE, core self-evaluation; CC, career calling; CDD, career decision-making difficulties.**

### Gender Differences

To further verify whether there were significant differences between male and female in the mediating model, we tested the structural equation model on two samples. The results showed that the model fit well for both male and female sample (Male: χ^2^/*df* = 3.851, RMSEA = 0.073, GFI = 0.943, NFI = 0.949; Female: χ^2^/*df* = 3.957, RMSEA = 0.071, GFI = 0.948, NFI = 0.929, see Supplementary Table 4).

Then, the structural path coefficient between variables was estimated freely (M2) under the condition that the measurement weights of both two groups of subjects were equal. Compared with the M1 without any restriction, the results revealed that the fitting degree of M2 was better (Δ NFI = 0.004, Δ IFI = 0.004), and a significant difference existed in M2 and M1 (*p* = 0.002). It showed that the model is unstable in male and female samples. On the basis of M2, the structure weight of the variables was restricted to be equal (M3). The results showed that the fitting index of M3 and M2 existed a significant difference (*p* = 0.005, Δχ^2^/Δ*df* = 2.32, ΔNFI = 0.004, ΔIFI = 0.004), suggested that there were considerable differences in this indirect effect model between male and female (see Table 2).

Path coefficient comparison was further performed, and found that there were considerable differences between male and female students on the four various paths: Respectively, the relationship between career calling and career decision-making difficulties was greater in male than in female (c. r. = –2.53, *p* < 0.05); the relationship between paternal positive parenting style and career decision-making difficulties was significant in male students, but absent in female students (c. r. = –3.05, *p* < 0.05); the relationship between maternal positive parenting and career decision-making difficulties was significant in female students, but absent in male students (c. r. = 2.52, *p* < 0.05); and the relationship between paternal negative parenting and career calling was significant in female students, but absent in male students (c. r. = –1.99, *p* < 0.05).

## Discussion

Based on a survey of 1,127 college students, this study was among the initial efforts in examining the associations between four facets of parenting styles on career decision-making difficulties as well as the mediating role of core self-evaluation and career calling in explaining such associations. The results largely supported that parenting styles were associated with career decision-making difficulties. Specifically, positive and negative paternal parenting styles had significant and direct predictive effects on career decision-making difficulties. Meanwhile, positive paternal and maternal parenting styles could predict career decision-making difficulties through career calling. Positive paternal parenting style and negative maternal parenting style also predicted career decision-making difficulties through core self-evaluation. In addition, core self-evaluation and career calling played a mediating role between positive paternal/negative maternal parenting styles and career decision-making difficulties. Furthermore, gender difference existed in the relationship that career calling predicted career decision-making difficulties, negative paternal parenting style predicted career calling, positive paternal parenting style predicted career decision-making difficulties, and positive maternal parenting style predicted career decision-making difficulties. To sum up, core self-evaluation and career calling played a mediating role in the relationship between parenting styles and career decision-making difficulties.

### Predictions of Parenting Styles for Career Decision-Making Difficulties

One of the most important findings of the current study was the directly predictive effect from positive and negative paternal parenting styles to career decision-making difficulties of college students. Specifically, positive paternal parenting style (emotional warmth) significantly predicted career decision-making difficulties, which consistent with previous studies (e.g., Hou et al., 2013; Wang, 2013). This result suggested that students who grow up in a family with warmth and understanding parents will experience less career decision-making difficulties. While previous research has consistently supported the role of positive parenting styles in promoting the career decision-making process (e.g., Koumoundourou et al., 2011; Liu, 2008), the current results shed additional light on the detrimental role of paternal emotional warmth in positively predicting career decision-making difficulties. Compared with mothers, fathers’ social status, professional quality and male characteristics have a special influence on their children’s growth (Sun and Li, 2015). Together with the previous research (Hou et al., 2013), individuals with more paternal emotional warmth were more likely to not only promote social ability and personality, but also make good career decisions. Thus, emphasizing the care and guidance of fathers on individuals can more directly help college students to successfully complete employment.

Moreover, the result showed strong positive prediction function for negative paternal parenting style to career decision-making difficulties, which was contrary to previous research (e.g., Ketterson and Blustein, 1997; Koumoundourou et al., 2011). This may be due to the role of the overprotective dimension of negative paternal parenting styles. The overprotective dimension, such as arrangement of work for the child directly or control of action, makes the student follow his planned career path without career exploration and feel more career decision-making self-efficacy (Liu, 2008). Moreover, after analysis of separate samples, this effect only existed in the female group, which further proves our hypothesis that father’s overprotective of female students makes them less likely to explore careers and have a low level of difficulty in career decision-making. This finding was important for a holistic understanding of the role of parenting styles in career decision-making difficulties, and has important implications for career interventions. However, it should be pointed out that this overprotection is not conducive to individual development. It can be seen that fathers’ participation in individual career decision-making should be strengthened and the important role of fathers in parenting practice should be emphasized in the process of individual career decision-making.

### The Mediating Roles of Core Self-Evaluation and Career Calling

The current study was particularly interesting in illuminating potential predictors of core self-evaluation and career calling. As expected, it revealed that the role of parenting styles on career decision-making difficulties can be realized through the bridge of core self-evaluation and career calling. More specifically, positive paternal parenting style and negative maternal parenting style could not only directly predict career decision-making difficulties, but also indirectly predict career decision-making difficulties through core self-evaluation and career calling. The positive parenting style of mothers indirectly predicted career decision-making difficulties through career calling. These predictive effects were consistent with results of prior studies (Hall and Chandler, 2005; Hou et al., 2013; Neubert and Halbesleben, 2015). Parents who adopt an emotionally warm parenting style usually provide more attention and encouragement and offer constructive guidance to their children, rather than leaving their overwhelmed children disoriented, which will promote their children’s self-confidence and problem-solving skills. That means, individuals who experienced warmth and support at home in career decision-making tend to have a higher level of confidence and self-efficacy, experience positive evaluation of themselves, develop a positive intuition and responsibility about work, and move rapidly and disengage easily from the decision-making process (e.g., Judge et al., 1997). Conversely, individuals who perceived their parents as exhibiting a strict control over their behavior tended to show less confidence in themselves and less concern for them jobs, thus showing more difficulties in their decision-making process. More importantly, it suggested that how people respond to parenting styles in career decision-making is potentially related to their attitudes toward careers and themselves. In the era of information sharing, teenagers can easily get information about careers, so as to have more autonomy in choosing careers and make career decision according to their interests or attitudes. Therefore, more attention should be paid to the support and encouragement of parents’ emotional warmth for teenagers, so that teenagers have the courage and confidence to make their desired career decisions.

The results also supported social cognitive career theory (Lent and Brown, 1996), which holds that in the process of career decision-making, environmental factors such as parenting styles and social support can indirectly influence the career decision-making process by influencing individual variables (such as career calling and core self-evaluation) which would to influence the goals and actions of career choice. Positive parenting style will make students experience more warmth and respects, increase their emotion positively, and make them have a sense of meaning and value beyond their own in terms of career decision (Dik et al., 2009). Thus, it can encourage students to care about their future career, so as to enhance their career calling. At the same time, parents’ positive parenting styles will cultivate their offspring’ self-esteem and self-confidence, improve their sense of professional self-efficacy, and make their self-evaluation higher (e.g., Xie, 2012). Individuals with parents emotional warmth will be more confident in their career choice, actively explore their career and reduce difficulties during their career decision-making processes (Fabio et al., 2012; Zhang, 2019). However, individuals brought up in a negative parenting environment seem to develop negative self-evaluations and lower expectations for work, which in turn make them to experience more difficulties in their career choice process. Based on this finding, we should pay attention to the importance of parenting styles, strengthen emotional warmth and reduce rejection and overprotection of parents in the process of career exploration. Under the synergistic effect with parents, individuals can reduce anxiety about career decisions and make better choices.

### Gender Difference

It should be pointed out that the multiple mediators found it has certain gender differences in this study. This is mainly reflected in the following factors.

Firstly, in the sample of male students, the degree of career calling predicts career decision-making difficulty is higher than that of female students. In the context of Eastern culture, researchers believe that career calling is more rooted in internal forces than driven by external forces (Zhang et al., 2014; Zhang et al., 2015). In addition, considering that in Oriental families, parents tend to emphasize male’s career achievements, making male’s career ambition higher than females. Driven by this career ambition, male tend to follow their internal voice and pay more attention to their inner achievements and development (Rose and Rudolph, 2006). This is consistent with the current social situation. Male have greater career aspirations and ambition, and more hope to make certain achievements in their future career. Therefore, although both male and female students’ career calling can predict career decision-making difficulties, male students’ level of prediction is higher.

Secondly, females who perceive their father as exhibiting a strict control and rejection over their behavior tended to report less career calling. This may be because female are more susceptible to external influences than male (Rose and Rudolph, 2006). And career calling is more rooted in internal forces than driven by external forces (e.g., Zhang et al., 2015). It shows that the over-protection of fathers makes female lack of exploration of careers, thus affecting their assessment and intuition of career values.

Thirdly, the relationship which positive paternal parenting style directly predicted career decision-making difficulties only exist in the sample of male, and the relationship which positive maternal parenting style directly predicted career decision-making difficulties only exist in the sample of female. In terms of positive parenting, “like-sex” plays an important role, that is, mother often participate more in the growth of their daughter, and son are often disciplined more by their father (Jan and Janssens, 1998; Liat, 2002). Combined with previous study (Liu et al., 2014), father is more involved in the upbringing of their son, and female have more dependence and communication with mothers. At least in this study, thus, like-sex plays a role of huge and direct. From the perspective of professional gender stereotypes, male gender stereotypes are stronger than female gender stereotypes (Liu and Zuo, 2006). In the traditional division of labor in a family where men work outside and women work inside, male are expected to work hard and female are expected to accompany their children more. The heritability of this idea makes the connections between fathers and sons, mothers and daughters stronger. Based on the findings, counselors could consider working with parents to consider like-sex relationship in parenting styles to enable individuals to reduce their anxiety about career decision-making and consequently reduce career decision-making difficulties and make adaptive career choices.

### Limitations and Implication for Future Research

In the present study, several limitations should be noted. First and foremost, the current study drew upon cross-sectional date to examine the dual mediator model. While the conceptual flow of this model was based on the temporal career decision-making difficulties, the temporal sequence and potential causality cannot be fully established without longitudinal date. To future consolidate the utility of parenting styles, it is necessary for future research to longitudinally validate the relationships in the current study. Then, as date in the current study was self-reported, some of the identified associations may be inflated. Future studies are warranted to employ multi-informant and multi-method designs to minimize shared informant. For example, parental styles can be reported by parents. Finally, the current study only examined three single pathways with gender differences. Future studies should further validate the differences between this dual mediating effect in the sample of male and female in order to better understand the potential role of parenting styles in predicting career decision-making difficulties.

## Conclusion

The conclusions can be drawn as follows: (1) positive and negative paternal parenting styles have a directly significant positive predictive effect on career decision-making difficulties in college students; (2) core self-evaluation and career calling play a chain-mediated role in the relationship that positive paternal parenting style predict career decision-making difficulties and negative maternal parenting style predict career decision-making difficulties; (3) gender differences exist in the process of parenting styles to predict career decision-making difficulties through core self-evaluation and career calling.

## Data Availability Statement

The raw data supporting the conclusions of this article will be made available by the authors, without undue reservation.

## Ethics Statement

The studies involving human participants were reviewed and approved by the Ethics Committee of Shandong Normal University. The patients/participants provided their written informed consent to participate in this study.

## Author Contributions

JS and DH conceived the idea and developed the materials. BH and HL carried out the data collection. XT and SX wrote and revised the manuscript. KA polished the language of this manuscript. JS, DH, BH, and HL provided critical feedback. All authors read and approved the final manuscript.

## Conflict of Interest

The authors declare that the research was conducted in the absence of any commercial or financial relationships that could be construed as a potential conflict of interest.
