# Supplementary material for: How Parenting Styles Link Career Decision-Making Difficulties in Chinese College Students? The Mediating Effects of Core Self-Evaluation and Career Calling
Source: Front Psychol. 2021 May 19;12:661600. doi: 10.3389/fpsyg.2021.661600 (PMC8172080; doi:10.3389/fpsyg.2021.661600)
Supplement: Supplementary file 1 [file Table_1.DOCX]

**Table S1** Regression analysis of parenting styles, core self-evaluation, career calling, and career decision-making difficulties

| Dependent variable | Predictor variable | *B* | *SE* | β | *t* |
| --- | --- | --- | --- | --- | --- |
| CSE | FPS-N | 0.03 | 0.08 | 0.17 | 0.34 |
|  | FPS-P | −0.23 | 0.04 | −0.20 | −5.43*** |
|  | MPS-N | 0.14 | 0.07 | 0.10 | 2.08* |
|  | MPS-P | −0.08 | 0.04 | −0.07 | −1.91 |
| CC | FPS-N | −0.04 | 0.04 | −0.06 | −1.06 |
|  | FPS-P | 0.04 | 0.02 | 0.08 | 2.00* |
|  | MPS-N | −0.05 | 0.03 | −0.08 | −1.53 |
|  | MPS-P | 0.11 | 0.02 | 0.24 | 5.67*** |
|  | CSE | −0.04 | 0.01 | −0.10 | −2.99** |
| CDD | FPS-N | 0.09 | 0.03 | 0.13 | 2.62** |
|  | FPS-P | 0.07 | 0.02 | 0.15 | 4.05*** |
|  | MPS-N | 0.002 | 0.03 | 0.003 | 0.05 |
|  | MPS-P | 0.008 | 0.02 | 0.02 | 0.44 |
|  | CSE | −0.10 | 0.01 | −0.23 | −7.99*** |
|  | CC | 0.46 | 0.04 | 0.45 | 12.43*** |

FPS -N = negative paternal parenting style; FPS-P = positive paternal parenting style; MPS-N = negative maternal parenting style; MPS-P = positive maternal parenting style; CSE = core self-evaluation; CC = career calling; CDD = career decision-making difficulties;

* *p* < 0.05; ** *p* < 0.01; *** p < 0.001. (same below)

**Table S2** The correlation analysis of parenting styles, core self-evaluation, career calling, and career decision-making difficulties of different gender college students

| variable | 1 | 2 | 3 | 4 | 5 | 6 | 7 |
| --- | --- | --- | --- | --- | --- | --- | --- |
| 1. FPS-N | - | −0.19** | 0.65** | −0.24** | 0.13** | −0.14** | 0.022 |
| 2. FPS-P | −0.06 | - | −0.14** | 0.60** | −0.28** | 0.22** | 0.25** |
| 3. MPS-N | 0.76** | −0.11* | - | −0.23** | 0.23** | −0.09* | 0.02 |
| 4. MPS-P | −0.22** | 0.69** | −0.20** | - | −0.26** | 0.28** | 0.28** |
| 5. CSE | 0.19** | −0.26** | 0.21** | −0.24** | - | −0.20** | 0.40** |
| 6. CC | −0.09* | 0.28** | −0.11* | 0.32** | −0.19** | - | −0.35** |
| 7.CDD | −0.05 | 0.37** | −0.10* | 0.29** | 0.53** | −0.31** | - |
| M _male_ | 26.21 | 19.08 | 27.20 | 20.67 | 21.11 | 39.84 | 56.36 |
| SD _male_ | 7.18 | 4.43 | 7.24 | 4.64 | 5.31 | 7.16 | 10.57 |
| M _female_ | 22.92 | 19.49 | 24.27 | 21.71 | 21.76 | 41.00 | 55.47 |
| SD _female_ | 5.82 | 4.81 | 6.21 | 4.67 | 5.01 | 5.91 | 8.57 |
| t | 8.51*** | −1.48 | 7.31*** | −3.74*** | −2.11* | −2.98** | −0.19 |

The coefficient below diagonal line is male; the coefficient above diagonal line is female.

**Table S3** Structural equation models for male and female group

| Dependent variable | Predictor variable | Male | | Female | |
| --- | --- | --- | --- | --- | --- |
|  |  | β | *t* | β | *t* |
| CSE | FPS-N | 0.12 | 1.53 | −0.01 | −0.15 |
|  | FPS-P | −0.22 | −3.78*** | −0.20 | −3.98*** |
|  | MPS-N | 0.05 | 0.69 | 0.12 | 2.08* |
|  | MPS-P | −0.04 | −0.70 | −0.11 | −2.09* |
| CC | FPS-N | 0.07 | 0.80 | −0.14 | −2.05* |
|  | FPS-P | 0.09 | 1.43 | 0.06 | 1.02 |
|  | MPS-N | −0.16 | −1.95 | 0.01 | 0.08 |
|  | MPS-P | 0.23 | 3.71*** | 0.24 | 4.05*** |
|  | CSE | -0.13 | −2.89** | −0.10 | −2.13* |
| CDD | FPS-N | 0.05 | 0.68 | 0.15 | 2.51* |
|  | FPS-P | 0.26 | 4.90*** | 0.07 | 1.46 |
|  | MPS-N | −0.03 | −0.04 | 0.03 | 0.61 |
|  | MPS-P | −0.09 | −1.59 | 0.11 | 2.04* |
|  | CSE | −0.17 | −4.27*** | −0.28 | −6.61*** |
|  | CC | 0.50 | 10.33*** | 0.37 | 7.08*** |

Note: * p < 0.05, ** p < 0.01, *** p < 0.001.

**Table S4** Indirect effect

| Sample | | Path | Indirect effect | | | Percentage of total effect |
| --- | --- | --- | --- | --- | --- | --- |
| Male | FPS-P→CSE→CDD | | | 0.020 | 10.53% | |
|  | FPS-P→CSE→CC→CDD | | | 0.008 | 4.21% | |
|  | MPS-P→CC→CDD | | | 0.061 | 54.95% | |
| Female | FPS-P→CSE→CDD | | | 0.023 | 34.85% | |
|  | FPS-P→CSE→CC→CDD | | | 0.003 | 4.55% | |
|  | FPS-N→CC→CDD | | | −0.038 | 24.84% | |
|  | MPS-P→CSE→CC→CDD | | | 0.002 | 2.02% | |
|  | MPS-P→CC→CDD  MPS-P→CSE→CDD | | | 0.038  0.013 | 38.38%  13.13% | |
|  | MPS-N→CSE→CDD  MPS-N→CSE→CC→CDD | | | −0.022  −0.003 | 45.83%  6.25% | |

In the sample of male students (see Fig. S1), core self-evaluation (β = −0.17, *p* < 0.001) and career calling (β = 0.50, *p* < 0.001) had significant predictive effects on career decision-making difficulties, while core self-evaluation was found to (negatively) predict career calling (β = −0.13, *p* < 0.01) (see Table S3 and Fig. S1 ); Positive paternal parenting style was found to predict core self-evaluation (β = −0.22, *p* < 0.001) and career decision-making difficulties (β = 0.26, *p* < 0.001), which showed that positive paternal parenting style could directly predict career decision-making difficulties in male students, and also could indirectly predict their career decision-making difficulties through core self-evaluation and career calling, the indirect effect was 0.028, accounting for 14.74% of the total effect (see Table S4). Positive maternal parenting style only had a significant predictive effect on male's career calling (β = 0.23, *p* < 0.001), and had no significantly and directly predictive effect on male's career decision-making difficulties in the presence of mediating variables. It showed that positive maternal parenting style could indirectly predict career decision-making difficulties through career calling in male students. The indirect effect was 0.061, accounting for 54.95% of the total effect (see Table S4). Negative maternal parenting style and negative paternal parenting style had no predictive effect on career calling, core self-evaluation, and career decision-making difficulties (*p*s > 0.05) (see Fig. S1).

In the sample of female students (see Fig. S2, Table S3), core self-evaluation (β = −0.28, *p* < 0.001) and career calling (β = 0.37, *p* < 0.001) were found to predict career decision-making difficulties, while core self-evaluation was found to (negatively) predict career calling (β = −0.10, *p* < 0.05); Positive paternal parenting style only had a significant predictive effect on core self-evaluation (β = −0. 20, *p* < 0.001), while it had no significantly and directly predictive effect on female’s career decision-making difficulties. It showed that positive paternal parenting style indirectly predicted career decision-making difficulties of female through core self-evaluation and career calling, and the indirect effect was 0.026, accounting for 39.40% of the total effect (see Table S4); Negative paternal parenting style was found to significantly predict female's career calling (β = −0.14, *p* < 0.05) and career decision-making difficulties (β = 0.15, *p* < 0.05), suggesting that paternal negative parenting style indirectly predicted female's career decision-making difficulties through career calling. The indirect effect was −0.038, accounting for 24.84% of the total effect (see Table S4); Positive maternal parenting style was found to predict female's core self-evaluation (β = −0.11, *p* < 0.05) , career calling(β = 0.24, *p* < 0.001) and career decision-making difficulties (β = 0.11, *p* < 0.05), suggesting that positive maternal parenting style could not only directly predicted female's career decision-making difficulties, but also indirectly predicted career decision-making difficulties through core self-evaluation and career calling. The indirect effect was 0.053, accounting for 53.53% of the total effect (see Table S4); Negative maternal parenting style only significantly predicted core self-evaluation (β = 0.12, *p* < 0.05). In the presence of core self-evaluation, the direct prediction effect on female's career decision-making difficulties was not significant. It was indicated that negative maternal parenting style could indirectly predict female's career decision-making difficulties through core self-evaluation and career calling, and the indirect effect was -0.025, accounting for 52.08% of the total effect (see Table S4).


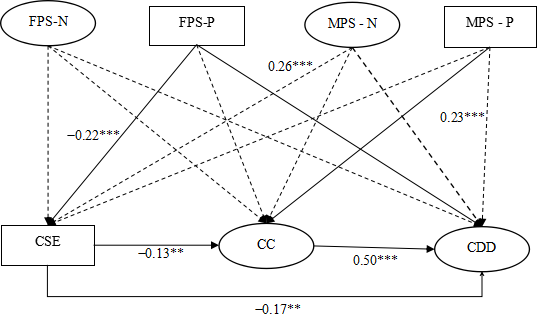


**Fig. S1** Path analysis of parenting styles and career decision-making difficulties (Male)


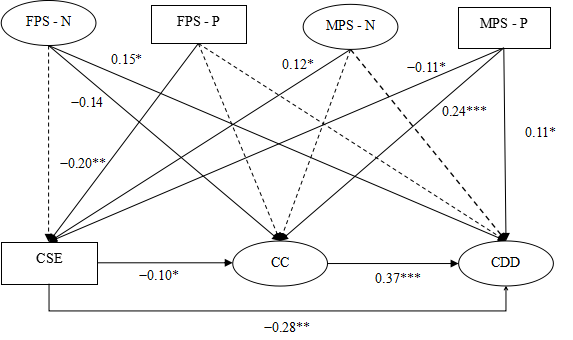


**Fig. S2** Path analysis of parenting styles and career decision-making difficulties (Female)
